# Seasonally Changing Cryptochrome 1b Expression in the Retinal Ganglion Cells of a Migrating Passerine Bird

**DOI:** 10.1371/journal.pone.0150377

**Published:** 2016-03-08

**Authors:** Christine Nießner, Julia Christina Gross, Susanne Denzau, Leo Peichl, Gerta Fleissner, Wolfgang Wiltschko, Roswitha Wiltschko

**Affiliations:** 1 Fachbereich Biowissenschaften der Goethe-Universität Frankfurt, Max-von-Laue-Str. 13, D-60438, Frankfurt am Main, Germany; 2 Haematology and Oncology and Developmental Biochemistry, University Medicine Göttingen, Justus-von-Liebig Weg 11, 37077, Göttingen, Germany; 3 Max Planck Institute for Brain Research, Max-von-Laue-Str. 4, D-60438, Frankfurt am Main, Germany; Lund University, SWEDEN

## Abstract

Cryptochromes, blue-light absorbing proteins involved in the circadian clock, have been proposed to be the receptor molecules of the avian magnetic compass. In birds, several cryptochromes occur: Cryptochrome 2, Cryptochrome 4 and two splice products of Cryptochrome 1, Cry1a and Cry1b. With an antibody not distinguishing between the two splice products, Cryptochrome 1 had been detected in the retinal ganglion cells of garden warblers during migration. A recent study located Cry1a in the outer segments of UV/V-cones in the retina of domestic chickens and European robins, another migratory species. Here we report the presence of cryptochrome 1b (eCry1b) in retinal ganglion cells and displaced ganglion cells of European Robins, *Erithacus rubecula*. Immuno-histochemistry at the light microscopic and electron microscopic level showed eCry1b in the cell plasma, free in the cytosol as well as bound to membranes. This is supported by immuno-blotting. However, this applies only to robins in the migratory state. After the end of the migratory phase, the amount of eCry1b was markedly reduced and hardly detectable. In robins, the amount of eCry1b in the retinal ganglion cells varies with season: it appears to be strongly expressed only during the migratory period when the birds show nocturnal migratory restlessness. Since the avian magnetic compass does not seem to be restricted to the migratory phase, this seasonal variation makes a role of eCry1b in magnetoreception rather unlikely. Rather, it could be involved in physiological processes controlling migratory restlessness and thus enabling birds to perform their nocturnal flights.

## Introduction

Cryptochromes are blue-light absorbing proteins found in bacteria, plants and animals. They play an important role in circadian clocks, in part as photoreceptors for entrainment; in plants, they control developmental processes like hypocotyl elongation and photoperiodic induction of flowering (for review, see [1,2]. In birds, they have also been proposed as receptor molecules for the magnetic compass, forming the crucial radical pairs that interact with the magnetic field. The Radical Pair Model of magnetoreception [3] suggests that the singlet/triplet ratio, which depends on the alignment of the radical pair in the ambient magnetic field, leads to an activation pattern on the retina that is centrally symmetric to the field lines and thus can provide the birds with information on magnetic directions.

In birds, cryptochromes were first discovered in the retina of chickens, *Gallus gallus*: cryptochrome 1 by Haque and colleagues [4], cryptochrome 2 by Bailey and colleagues [5], and more recently cryptochrome 4 by several groups [6–8]. They have been shown to respond to light [4,5, 8–11] and to be involved in the circadian system [12]. In the European robin *Erithacus rubecula* (Turdidae), a migratory passerine, Möller and colleagues [13] found two forms of cryptochrome 1, eCry1a and eCry1b, which are splice-products of the same gene, with different amino acid sequences at the C-terminus. By immuno-histological methods, Mouritsen and colleagues [14] identified cryptochrome 1 in the retinal ganglion cells, displaced ganglion cells and photoreceptors of the Garden Warbler *Sylvia borin*, also a migratory passerine, during migration. The antiserum used in that study, however, did not distinguish between the two splice-products of cryptochrome 1. Recently, we could identify Cry1a in the outer segments of the UV/V cones in the retinae of robins and chickens [10,15]. This raised the question whether the cryptochrome 1 described by Mouritsen et al. [14] in the retinal ganglion cells could be Cry1b. Hence the present study is devoted to the question where eCry1b is located in the retina of European Robins.

## Results

We studied the retinae of eight birds, five of them in migratory state when they showed nocturnal migratory restlessness, and three after their migratory period had ended (for details, see Methods section). There was a striking difference between the two groups.

**Fig 1:**
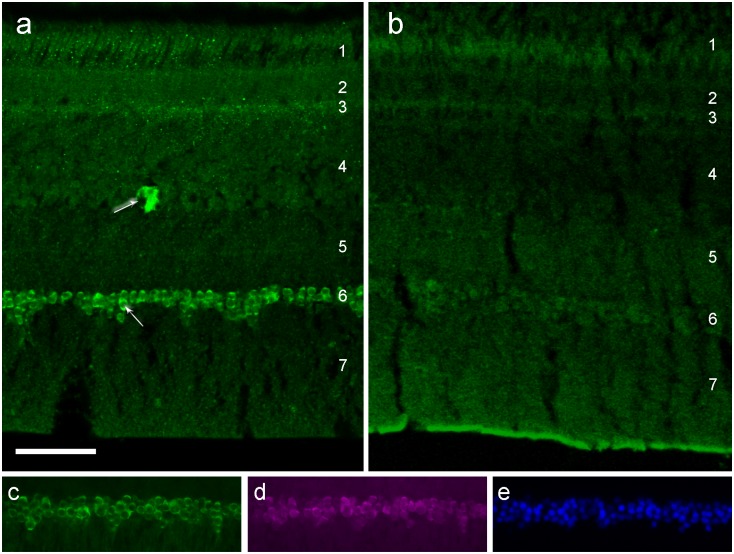
Immuno-labeling for eCry1b in the retina of European Robins. Retina of a robin (**a**) in migratory state, and of one (**b**) after the end of the migratory period. In (**a**), there is eCry1b labeling in the ganglion cells (layer 6) and the few displaced ganglion cells (layer 4). eCry1b label is located in the cytosol of the cell; the nuclei, indicated by arrows, show no label. In (**b**), the labeling in the ganglion cells is very low (see also Fig B in S1 Supporting Information). (**c-e**) Retinal ganglion cell layer in a robin in migratory state, triple-labeled for (**c**) eCry1b, (**d**) NeuN, and (**e**) DAPI. Practically all cells in the ganglion cell layer express eCry1b. Layers of the retina: 1, photoreceptor outer and inner segments; 2, outer nuclear layer; 3, outer plexiform layer; 4, inner nuclear layer; 5, inner plexiform layer; 6, ganglion cell layer; 7, optic nerve fibre layer. The scale bar is 50 μm for all panels.

In birds in migratory state, we found a considerable amount of eCry1b in all ganglion cells including the displaced ganglion cells (Fig 1a). The latter are not frequent, but rather regularly spaced in the inner nuclear layer, and they are markedly larger than the bipolar and amacrine cells in that layer. These displaced ganglion cells and the entire ganglion cell layer are clearly immuno-labeled by the antiserum against eCry1b. This was observed in robins during natural autumn migration, natural spring migration, and in a robin where spring migratory restlessness had been photo-periodically induced in early January ahead of time.

Counterstaining with an antibody against the neuron-specific marker NeuN showed that practically all ganglion cell layer neurons express eCry1b in the robins that were in migratory state. These eCry1b- and NeuN-immunoreactive cells represent the large majority of cells in the ganglion cell layer visualized by the nuclear stain DAPI (Fig 1c–1e). eCry1b is present in the ganglion cells across the retina, as documented by the sections cut across the entire expanse of the retina (Fig A in S1 Supporting Information). However, as we did not label retinal wholemounts, we cannot exclude that small areas of retina lack eCry1b expression.

In contrast, the amount of eCry1b was clearly reduced after the end of the phase of migratory restlessness: we did not find any significant immuno-labeling in the ganglion cells (Fig 1b). The weak labeling in the inner segments of the retinal photoreceptors in Fig 1b probably is an artifact, because it also occurs in the respective control with pre-immune serum (see Fig B in S1 Supporting Information).

**Fig 2:**
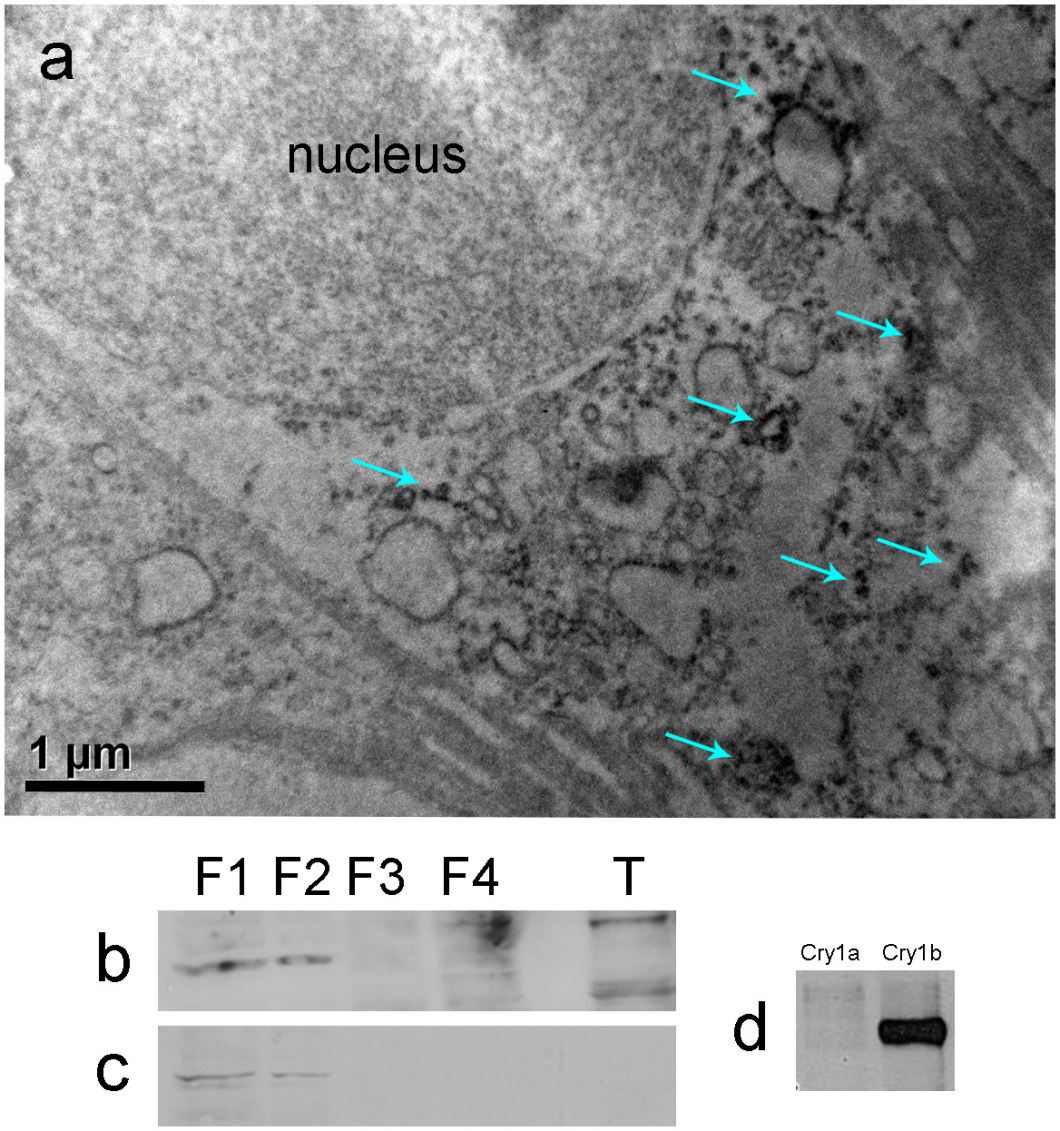
Electron-microscopic image of a ganglion cell, and Western blots. (**a**) Ganglion cell in the retina of a robin in migratory state. eCry1b labeling is visualized with diaminobenzidine and silver intensification, visible as dark dots (some marked by arrows). Other subcellular components cannot be identified; eCry1b is probably free in the cytosol and also bound to membranes. (**b**, **c**) Western blots of the robin retina, indicating eCry1b (**b**; ~65 kDa) and eCry1a (**c**; ~70 kDa) in the cytosolic and in the membrane fraction. Both cryptochromes were detected in the same blot; the part showing eCry1a was already published in [15]. F1, cytosolic fraction; F2, membrane fraction; F3; nuclear fraction; F4, cytoskeletal fraction; T, tongue tissue from the same bird as control. (**d**) Western blot of purified eCry1a and eCry1b that had been treated with the eCry1b antiserum as control for the specificity of the antiserum, indicating that there is no cross-reactivity of the eCry1b antiserum with eCry1a.

The light-microscopic image already suggests that eCry1b is present in the cytoplasm but not in the nucleus (see arrow in Fig 1a). This is also indicated by the immuno-labeling in the electron microscopic image in Fig 2a: eCry1b seems to occur only in the cytoplasm, probably free in the cytosol as well as bound to membranes (see also Fig C in S1 Supporting Information). Immuno-blotting of eCry1b in differentially fractionated cell lysates from robin retina supports this view: eCry1b is found in the cytosolic and in the membrane fraction (Fig 2b). Western blotting of purified eCry1a and eCry1b showed that the eCry1b antiserum used here is specific for eCry1b, i.e. there is no cross-reactivity with eCry1a (Fig 2d).

## Discussion

We found eCry1b located in the ganglion cells and displaced ganglion cells in the retinae of European Robins that were in migratory state. Here, our findings are in agreement with the results of Mouritsen and colleagues [14] in garden warblers: the labeling of the ganglion cells with a general antiserum against cryptochrome 1 that they discuss in their paper appears to mark Cry1b (see also [16]). A difference is that we did not find eCry1b label in any cells of the inner nuclear layer except in the few displaced ganglion cells. This could be a specific effect of the general antiserum against cryptochrome 1 or a species-specific difference. Mouritsen and colleagues [14] also briefly mention an expression of cryptochrome 1 in the photoreceptor layer, which probably is the Cry1a that we later found located in the outer segments of the UV/V-cones [15].

### Comparison between eCry1b and eCry1a

Western-blotting showed eCry1b in the cytosol and in the membrane fraction, like eCry1a that we had identified earlier [15]. The antiserum used here is specific for eCry1b; it does not cross-react with eCry1a. This was to be expected, because the antiserum labels an epitope at the C-terminus of eCry1b that is different from the respective part of eCry1a, the other splice product of the same gene. It is also evident from the immuno-histochemical images: eCry1b label is absent from the photoreceptors where we had found Cry1a [15].

The position of the binding site of the eCry1b is unclear. This is also true for eCry1a which also occurs bound to membranes [15]. The most parsimonious assumption is that the binding site is the same in both splice variants. This would mean, however, that it does not lie in the region of the C-terminus, because that is where the two proteins differ. Also, in Cry1a, the C-terminus appears to be normally hidden and becomes only accessible when the protein is activated by light [10].

### A Function in Magnetoreception?

A characteristic of cryptochromes is that they form radical pairs when excited by light. Because of this, they were proposed as potential receptor molecules for sensing magnetic directions in the Radical Pair Model of magnetoreception [3]. Mouritsen and colleagues [14] discussed their findings in this context, suggesting that the cryptochrome 1 in the displaced ganglion cells as putative receptor molecule that forms the crucial radical pairs. They compared their data from garden warblers with those from zebra finches, *Taeniopygia guttata* (Estrildidae), possibly assuming that these birds, being non-migrants, did not have a magnetic compass. In their zebra finch samples, they indeed did not find any labeled ganglion cells at night and only few weakly labeled ones during the day [14].

However, magnetic compass orientation is not restricted to avian migrants. It was reported for the non-migratory homing pigeon [17,18] and, by conditioning experiments, it has since been demonstrated in two further non-migratory avian species, the domestic chicken [19] and the zebra finch [20]. Experiments with radio frequency fields, a diagnostic tool for radical pair mechanisms [21,22], indicated that the latter two species have the same type of magnetic compass based on radical pair processes as robins [23,24]. Orientation by a magnetic compass thus seems to be a general ability of birds, used for orientation within the home range (see [25]); it does not seem to be in any way restricted to migration.

In view of this, the findings of Mouritsen and colleagues [14] and our present findings do not necessarily support a role of Cry1b in magnetoreception, because the receptor molecule for magnetic directions should be present all year round. Magnetic information appears to be transmitted in the visual system channels for further processing in the central nervous system (see, e.g., [26, 27]). Of the two Cry1 splice products present in the retina, Cry1a seems to be better suited. It is expressed independent of the season and of the time of day in the outer segments of the UV/V cones in robins and chickens, and it fulfils all requirements of the Radical Pair Model [3], as the Cry1a-containing UV/V cones are distributed all across the retina, representing all spatial directions. Within these cones, the molecules are located along the disk membranes in a more or less regular arrangement [15] so that within any one cone cell they are oriented in about the same direction, and their responses can add.

### A Role in Circannual and Circadian Control?

We can only speculate about the possible function of eCry1b. Haque et al. [4] reported a larger of amount of Cry1 mRNA in the photoreceptor layer and in the ganglion cell layer of domestic chickens, showing a rhythmic expression. The former is probably associated with the Cry1a located there, the nature and function of the one in the ganglion cells is unclear. A circadian oscillation of the eCry1b protein in the retina is not indicated by our data, neither in migratory nor in non-migratory robins—there were no differences in the staining pattern associated with the time of day at which the birds were sacrificed (see Table 1). However, we cannot exclude that Cry1b plays a different role in migrants and non-migrants, and/or in passerine and non-passerine birds. A more detailed analysis of potential circadian effects was beyond the scope of the present study. Yet proteins involved in circadian rhythm control mostly act on nuclear transcription and hence are localized in or near the nucleus. This also applies to cryptochromes acting in the circadian negative feedback loop [28, 29]. However, the present data show a cytoplasmatic localization of Cry1b in the immunolabel and the Western blots, as do the data of Mouritsen et al. [14].

**Table 1:** Robins used in this study.

Birds	Date	Time	Migratory State
R 1	9 Jan.	17:30**	spring migratory activity *
R 2	6 April	19:00	no longer migratory activity *
R 3	6 April	12:00	no longer migratory activity *
R 4	11 April	17:30	no longer migratory activity *
R 5	21 April	13:45	natural spring migration
R 6	29 Sept.	18:30	natural autumn migration
R 7	3 Oct.	11:00	natural autumn migration
R 8	18 Oct.	07:00	natural autumn migration

Time is given in Central European Time (UTC+1).

* These birds had been photo-periodically manipulated (see Methods section) to induce premature spring Zugunruhe in early January.

** 17:30 corresponds to a subjective time for the birds of 19:30.

The seasonal change in the eCry1b expression reported here, together with the almost entire lack of expression in non-migratory zebra finches suggests that it could play a role in the physiological processes that control migration. Both garden warblers and European robins migrate at night; in view of the general involvement of cryptochromes in circadian rhythmicity (e.g.[1,2]), Cry1b might even take part in the shift in activity into the night for the nocturnal migration flights, as suggested by the results of Mouritsen et al. [14] and Fusani et al. [11]. Further studies are needed to clarify the true function of Cry1b in the various avian species.

## Methods

### Ethics Statement

Collecting of the birds was authorized by the Untere Naturschutzbehörde (ethics and natural protection committee) of the City of Frankfurt am Main; permit 7922–1.62-EA 07–0005. Four birds had been kept over the winter in single cages and tested during spring migration in behavioral tests following the normal routine (see, e.g., [30, 31]) under the permissions VI 63 – 19c 20/15 –F104/41 and V54 – 19c 20/15 –F104/47 issued by the Regierungspräsidium Darmstadt (Regional Administrative Authority).

### Birds

We used the retinae of eight European Robins for this study. The birds had been caught as transmigrants in the Botanical Garden of Frankfurt University in September and early October, except for one bird that killed itself by flying against a window on 21 April. They were identified as probably belonging to the migrating Scandinavian population by their wing lengths. Three birds were sampled immediately after they had been caught in autumn; these birds and the one obtained in April were in their natural migratory state. The four other birds had been used in orientation experiments (for details on housing and feeding, see Text A in S1 Supporting Information). To induce premature ‘Zugunruhe’ (migratory restlessness) in the beginning of January, they were photo-periodically manipulated: until December, they were kept under a decreasing photoperiod simulating the natural one down to L:D 8:16; around New Year, light was prolonged in two steps to L:D 13:11. This caused the birds to exhibit nocturnal Zugunruhe and oriented behavior towards North (see e.g. [30, 31]). One bird was taken a few days after the beginning of the orientation tests; it was in an artificially induced migratory state. The three remaining robins were sampled in the beginning of April, after the pre-seasonal Zugunruhe had ended and some of the birds had been laying eggs (see Table 1 for details).

### Tissue Preparation

Birds were killed by an overdose of Narcoren (Merial GmbH, Hallbergmoos, Germany). For both light and electron microscopy, retinae were fixed in the eyecup with 4% paraformaldehyde (PFA) in 0.1 M phosphate buffered saline (PBS, pH 7.4), or alternatively in 0,25% GA + 2% PFA in cacodylate buffer for 4 h at RT; then the PFA was washed out with PBS or cacodylate buffer (pH 7.4), respectively. For light microscopic immuno-histochemistry, retinae were cryoprotected in an ascending series of sucrose solutions (10%, 20%, 30% in PBS), and embedded in the tissue freezing medium Tissue-Tek (Sakura). 12–15 μm transverse sections were cut on a cryostat and mounted on Super Frost Plus slides. The slides were stored at –20°C until further processing.

For electron microscopy, after fixation and washing in PBS or cacodylate buffer, respectively, the retinae were embedded in agarose, and 60–100 μm sections were cut with a vibratome. The sections were treated free floating. They were first incubated in sucrose (10%, 20%, 30%), frozen three times in liquid nitrogen and then stored at –20°C until use. For cell fractionating and Western blot analysis, retinae were directly disintegrated to the F1 buffer according to the ProteoExtract^®^ Subcellular Proteome Extraction Kit manual (Calbiochem).

### Antibodies

A rabbit anti-eCry1b antiserum was designed in our laboratory and produced by GENOVAC GmbH, Freiburg, Germany; it was raised against amino acids 577–587 of eCry1b: CNYGK PDKTS K. Western blotting showed that the antiserum is specific for eCry1b in robins (Fig 2b), and that it does not cross-react with Cry1a (Fig 2d), see [16]. A commercial monoclonal antibody against NeuN (Neuronal Nuclei; clone A60, Merck Millipore MAB377) was used to label the retinal neurons [32, 33].

### Light Microscopic Immuno-Histochemistry

For light microscopic immuno-histochemistry, retinal sections on the slide were pre-incubated with 10% normal donkey serum (NDS) in 0.25% Triton X-100, 2% BSA in PBS for 60 min at RT. Then the sections were incubated with the primary antibodies (eCry1b 1:100; NeuN 1:500) in 3% NDS, 0.25% Triton X-100, 2% BSA, in PBS overnight at 4°C. After washing in PBS, the tissue was incubated with a donkey anti-rabbit secondary antibody coupled to the fluorescent dye Rhodamine Red-X, Alexa488 or Cy5 (Dianova, Hamburg) in 3% NDS, 0.25% Triton X-100, 2% BSA, in PBS for 1h at RT. After staining, the sections were coverslipped with Aqua—Poly Mount (Polysciences Europe), or with Vectashield Mounting Medium with DAPI to label all cell nuclei. Staining was evaluated with a confocal laser-scanning microscope (Zeiss Typ 510 META) or an epiflourescence microscope (Zeiss Axioplan 2). In the images fluorescence label was rendered in false colors.

Several controls were performed to show the specificity of immuno-labeling. A first control with pre-immune serum taken before immunizing the animals showed that there were no unspecific tissue reactions to other antibodies that were already present in the animals before immunization. The second control omitted the primary antibody from the above protocol, showing that the secondary antibody reacted selectively with the primary antibody and produced no artifacts. The third control was performed with the eCry1b antibody and the specific peptide that was used to produce the antibody. Before applying the primary antiserum on the retina, it was blocked by mixing it with this peptide. Here, any remaining label would indicate that the eCry1b antibody additionally recognizes other epitopes than the immunizing peptides, or that there are other antibodies in the serum that also bind to retinal structures. This was not the case. The controls are shown in Fig D in S1 Supporting Information.

### Pre-Embedding Immuno-Electronmicroscopy

After pre-incubation in 10% normal goat serum (NGS) and 2% bovine serum albumin (BSA) in PBS for 60 min at RT, retinal vibratome sections were incubated with the primary antibody (1:100) in 3% NGS, 2% BSA, in PBS over 3–4 days at 4°C. The secondary antibody was a biotinylated goat anti-rabbit IgG (Vector laboratories) applied for 2 hours. Then a peroxidase-based enzymatic detection system (Vectastain Elite ABC kit; Vector) was used. For visualizing the antibody bindings, the sections were treated with 0.025% diaminobenzidine for 15 minutes. For amplification of the immune signal, a silver intensification was used [34] (Sassoe-Pognetto et al. 1994). The sections were incubated in 0.5% osmium tetroxide for 30 minutes at 4°C, dehydrated by an ethanol series and propylene oxide and embedded in Agar Low Viscosity Resin (Plano GmbH, Agar Scientific Limited, Essex). Ultrathin sections (50–60 nm) were cut with an Ultra S microtome (Reichert, Leica) and placed on copper grids, stained with uranyl acetate and lead citrate and evaluated with a transmission electron microscope (CM12, Philips, Hamburg). Here we also performed controls with pre-immune serum and controls without the primary antibody.

### Western Blot and Cell Fractionation

The Western Blots used here are the same as the ones used in [15]. Cell fractionation was performed with the ProteoExtract^®^ Subcellular Proteome Extraction Kit (Calbiochem) according to the manufacturer’s manual. Then 20 μg of protein sample were subjected to 10% SDS-polyacrylamide gel electrophoresis and electro-blotted onto nitrocellulose membrane for 2 hours at 180 mA. After blocking with 5% BSA, the membranes were incubated with the rabbit eCry1b antiserum (1:500), followed by horseradish peroxidase-conjugated goat anti-rabbit IgG polyclonal antiserum (Dianova, Hamburg, Germany). Immunoblots were visualized using a solution of 2.5 mM luminol, 0.4 mM p-coumaric acid, 100 mM Tris-HCl, pH 8.5 and freshly added 0.009% H_2_O_2_. Tongue of the respective birds, a tissue without cryptochrome, was used as a control.

## Supporting Information

S1 Supporting Information Additional Figures and Text. (PDF)
