# Supplementary material for: Seasonally Changing Cryptochrome 1b Expression in the Retinal Ganglion Cells of a Migrating Passerine Bird
Source: PLoS One. 2016 Mar 8;11(3):e0150377. doi: 10.1371/journal.pone.0150377 (PMC4783048; doi:10.1371/journal.pone.0150377)
Supplement: S1 Supporting Information — (PDF) [file pone.0150377.s001.pdf]

S1 Supporting Information

for "Seasonally changing cryptochrome 1b expression in the retinal ganglion cells of a migrating passerine bird" by Christine Nießner, Julia Christina Gross, Susanne Denzau, Leo Peichl, Gerta Fleissner, Wolfgang Wiltschko, Roswitha Wiltschko

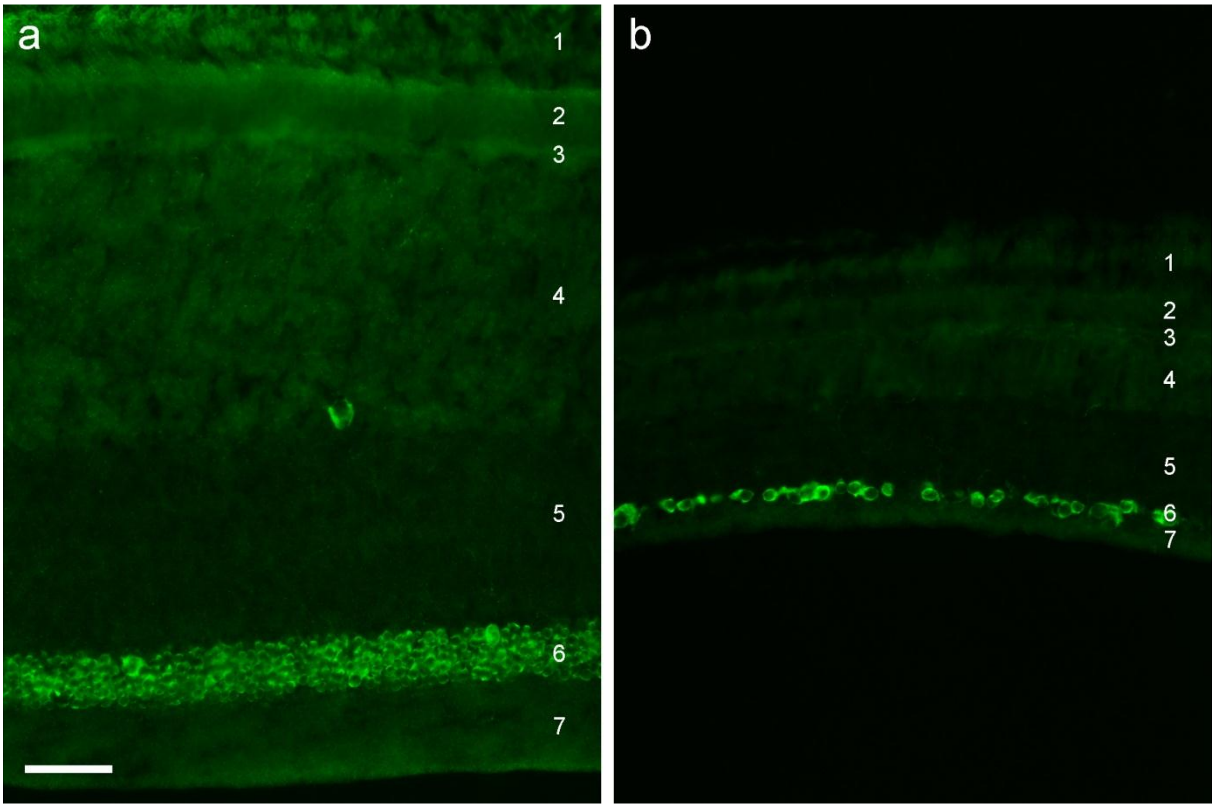

Figure A. eCry1b immuno-label is present across the robin retina. The labeling pattern across the retina of a robin in migratory state shows that the ganglion cells in **(a)** central and **(b)** peripheral retina are labeled. The density of ganglion cells and the overall thickness of the retina decrease from center to periphery. In (a), there is one displaced ganglion cell labeled in the inner nuclear layer near the border to the inner plexiform layer.
Layers of the retina: 1, photoreceptor outer and inner segments; 2, outer nuclear layer; 3, outer plexiform layer; 4, inner nuclear layer; 5, inner plexiform layer; 6, ganglion cell layer; 7, optic nerve fiber layer. The scale bar is 50 µm for both panels.

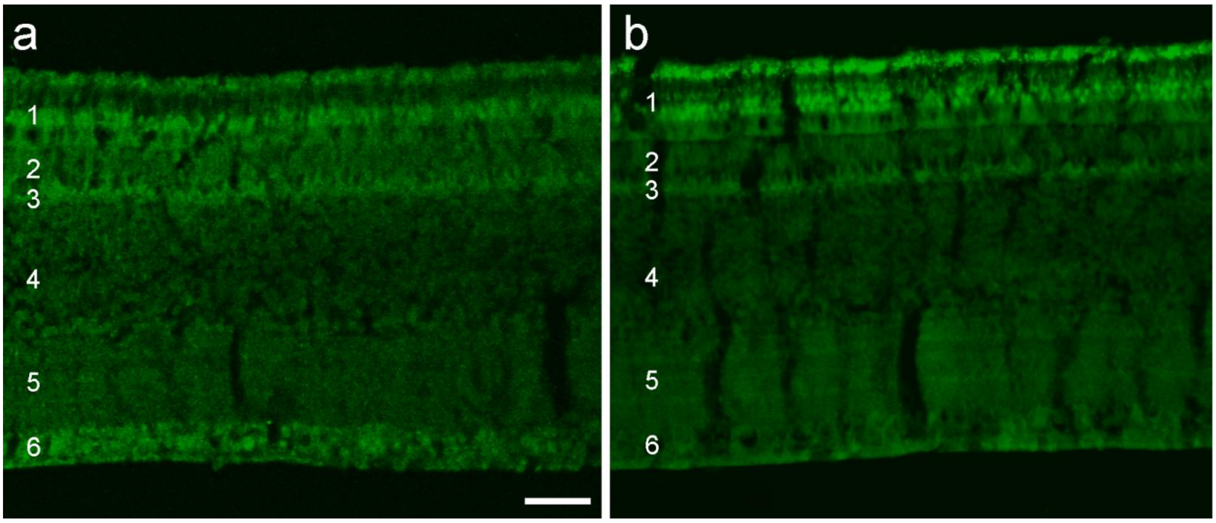

Figure B. Pre-immune serum control and eCry1b labeling in the retina of a non-migratory robin. (**a**) eCry1b immuno-labeling of the contralateral retina of the bird shown in Fig. 1B, indicating weak labeling of ganglion cells and some labeling of the inner segments of the photoreceptors. (**b**) Control slide treated with pre-immune serum, indicating that the labeling of the inner segments is non-specific. The signal is higher than in the immuno-histological staining with the antibody, because the pre-serum, unlike the antibody, is not purified. – Layer numbers and scale as in Fig. A.

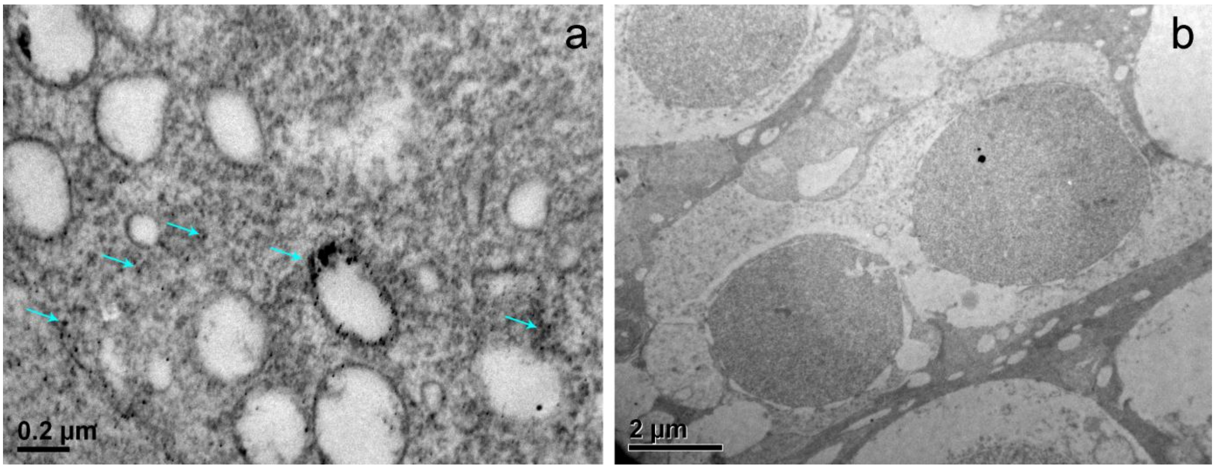

Figure C. Higher power electron micrograph of eCry1b immuno-labeling and pre-immune serum control. (**a**) Cytoplasm of a robin retinal ganglion cell. eCry1b labeling is visualized with diaminobenzidine and silver intensification, visible as dark dots (some marked by arrows). (**b**) Control section treated with pre-immune serum, no labeling is seen.

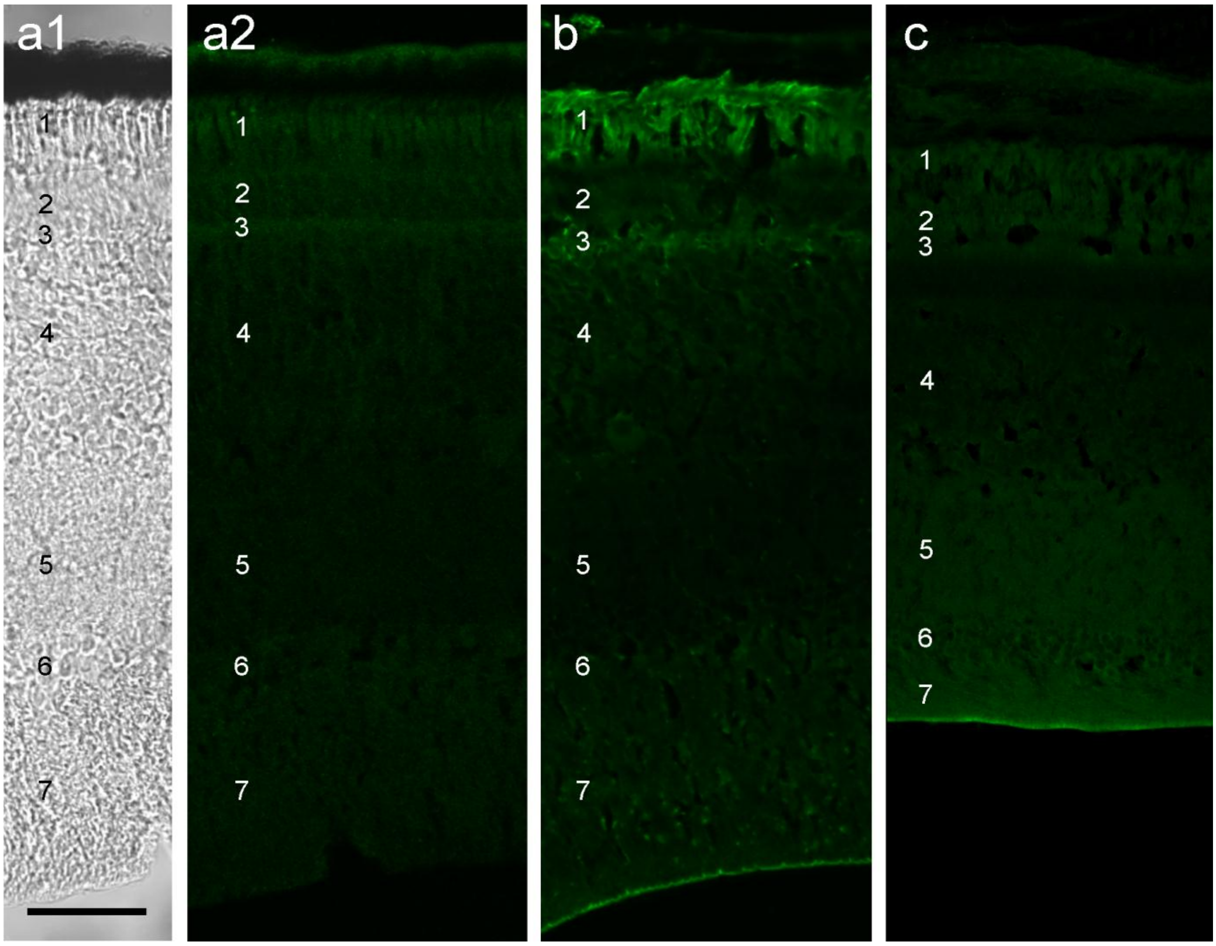

Figure D. Control of specificity of the secondary antibody; pre-immune serum and peptide control for anti-eCry1b in a robin retina. (**a1**) phase contrast image of section a2 indicating the various layers, layer numbers as in Fig. S1. (**a2**) Tissue incubation with only the secondary antibodies (omission of the primary antibodies from the protocol) showing that these antibodies reacted selectively with the primary antibodies. (**b**) Control with pre-immune serum, indicating that there was no unspecific reaction by other antibodies already present in the immunized animals, except for some reactivity in the photoreceptors (see Fig. S1B). (**c**) Control with the antibody and the specific peptide used to produce the antibody. The primary antiserum was blocked by mixing it with this peptide before applying it to the retina. Here, any remaining label would indicate the presence of other antibodies in the serum that also bind to retinal structures, which evidently is not the case. - Layers of the retina and scale bar as in Fig. A.

Text A. Bird housing and care

The birds that were kept over the winter and used for orientation experiments (see Wiltschko et al. 2014) lived in individual cages, ca. 60 x 30 x 40 cm, made from plastic with metal bars in front, equipped with two wooden perches of different diameters and two attached plastic cubes, one for water and one for food. The food consisted of a commercial food mixture for robins and thrushes (Aleckwa) with egg food for canaries, a calcium mixture and liquid vitamins added. It was meliorated by mixing it with grated apples or carrots or both and supplemented by mealworms. A fresh portion of this food was given daily, together with fresh water.

The robins adjusted quickly to the life in the cage, and the behavioral testing – placing them for 1 h into a funnel-shaped test cage – was associated with only little stress once it became routine, and with no pain at all. Further actions to minimize potential pain were not necessary.

Reference:

Wiltschko R, Gehring D, Denzau S, Nießner C, Wiltschko W. Magnetoreception in birds: II. Behavioural experiments concerning the cryptochrome cycle. J Exp Biol. 2014; 217: 4225-4228.
